# Does diagnostic delay of colorectal cancer result in malpractice claims? A retrospective analysis of the Swedish board of malpractice from 1995–2008

**DOI:** 10.1186/1754-9493-6-13

**Published:** 2012-06-18

**Authors:** Larsolof Hafström, Henry Johansson, Jon Ahlberg

**Affiliations:** 1 Department of Surgery, Sahlgrenska University Hospital, Göteborg, Sweden and the Swedish Patient Claims Panel, Stockholm, Sweden; 2 Department of Surgical Sciences, University Hospital, Uppsala, Sweden and the Swedish Patient Claims Panel, Stockholm, Sweden; 3 The County Council´s Mutual Insurance Company, Stockholm, Sweden

**Keywords:** Colorectal cancer, Diagnostic delay, Insurance claim review, Medical errors, Patient safety

## Abstract

**Aim:**

Delay in the diagnosis of colorectal cancer (CRC) may have important clinical and medico-legal implications. This study identifies the claims made on the basis of delay in the diagnosis of CRC to the Swedish insurance agency (whose English name is The County Council´s Mutual Insurance Company) and the impact and consequences of the delay on prognosis, treatment and survival for patients who reported the claims. The Company handles claims of medical malpractice where claimants seek compensation for alleged suffering and/or negative clinical impacts of diagnostic delays.

**Material and methods:**

Between January 1, 1995 and December 31, 2008, a total of 80 patients filed claims for negative effects resulting from delays in the diagnosis of CRC. Review of the claims led to identification of delay for 62 patients. The clinical symptoms that were overlooked and other causes of delay that had any relation to therapy, prognosis and economic compensation were evaluated.

**Results:**

The median delay in the diagnosis of CRC was six months. This delay was considered to have had an impact on the therapy in 20 % of the cases**.** The prognosis was postulated to have been adversely affected for 15 % of the patients. The delay was mainly caused by incomplete consideration of the symptoms hematoschisis or anaemia, changed bowel routine, or incomplete clinical or radiological examination and by misinterpretations of the results. No impact of duration of delay on survival was identified. The importance of identifying concomitant metastatic disease at diagnosis was overwhelming. Economic compensation was given in 79 % of the cases.

**Conclusion:**

This study found that claims for compensation for delay in diagnosis of CRC are rare. The delay in the diagnosis of the primary tumour was considered to have had an impact on the magnitude of therapeutic measures for a fifth of the patients who filed claims. Economic compensation for the patients´ injuries was given in almost 80 % of the cases.

## Background

Health-care related injuries and adverse events are always harmful for patients. In international analysis the incidence of adverse advents varies between 2.9-16.6 % [1,2]. In a Swedish study it was assessed to be 12.3 % [3]. In Sweden, with a population of 9.3 million, the official number of patient injuries exceeds 10,000 per year corresponding to less than one percent of all hospitalizations [4]. Among adverse events, doctors’ delay in diagnosis and management of malignant diseases is well recognized.

**Figure 1:**
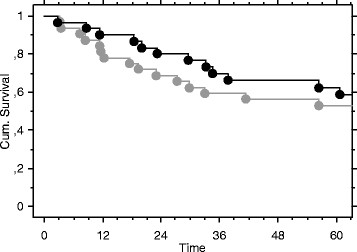
Survival curves (months) for patients with less than 6 months delay (black) and more than 6 months (gray).

**Figure 2:**
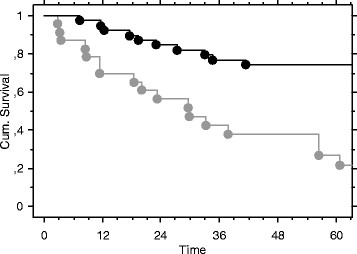
Survival curves (months) for patients without metastatic disease (black) and with metastatic disease (gray).

In Sweden colorectal cancer (CRC) is common and about 6200 individuals annually will get the diagnosis of CRC. In Sweden there are no available data about the consequences or impact of delay in diagnosis and treatment of CRC. A delay in the diagnosis of any cancer causes the patient to believe that she/he has lost the chance of cure or improvement. There are also undoubtedly deplorable psycho-social consequences associated with diagnostic delay. It is also important to consider any medico-legal implications of health-care related injuries and adverse events, which in the individual case can have very serious consequences.

Sweden has had a patient insurance system to compensate patients for health-related injuries since 1975. Patients who have been treated within the medical service system financed by the county medical councils (Landstinget in Swedish) can report their claims to the malpractice insurance review board referred to here as LÖF (Landstingens Ömsesidiga Försäkringsbolag, in translation to English The County Council´s Mutual Insurance Company). The patients may request economic compensation for the suffering and/or negative impacts on the treatment and the prognosis of the disease they have experienced. The insurance covers both physical and psychological injuries. The insurance company LÖF processes more than 90 % of all patient injury claims, mainly as a consequence of the county councils being responsible for almost (more than 90 %) all medical care in Sweden. In this health-care system it is uncommon for patients to request a second medical opinion.

The aim of this retrospective study was to identify the cause of the claims made to LÖF for diagnostic delay of CRC and to explore the consequences of the claimed delay on treatment, prognosis and survival. It also identified the extent to which economic compensation was given to the claimers.

## Material and methods

In Sweden (9,300,000 inhabitants) there are annually 6200 individuals who will get the diagnosis of CRC. Between January 1, 1995 and December 31, 2008 altogether 80 claims to LÖF were identified under the heading CRC-delayed diagnosis. Nine of the 80 claims could be excluded as they were related to treatment complications, six were registered under the wrong heading, and in three cases no cancer was diagnosed. The remaining 62 claims were recognized as involving delayed diagnosis and the charts and histopathological reports of these claims were scrutinized.

The alleged diagnostic delay was calculated in months from the date given in the patient´s report to LÖF to the date of clinically established diagnosis of cancer. Each author judged separately the impact of delay on prognosis and consequences on therapy without reference to the statements by the reviewers at LÖF. In the cases where there were differences between the authors´ judgments the terms supposedly or probably were added. There was no analysis of the psychological harm the delay could have caused the individual patient.

Data concerning survival were received from Swedish Cancer Register. Survival time was calculated from date of established diagnosis of cancer.

### Statistics

The survival probability was analyzed using the Kaplan-Meier method (Stat-view® 5.0). Anova factorial (Stat-view® 5.0) was used for analyzing differences between groups.

## Results

It was calculated that LÖF receives 4.4 claims dealing with CRC annually corresponding to 0.7 per 1000 new cases of CRC. Between 1995 and 2005 there were 62 claims of diagnostic delay of CRC. In 51 claims there was a delay in diagnosing the primary tumour and in two diagnosing recurrent/metastatic disease. In nine cases the delay was attributed to systemic errors made by the providers, in 7 delays in remitting the patient to the X-ray or CRC care unit. In one case the reply from the X-ray unit disappeared and in one an exchange of X-ray answers was found. In 34 cases the diagnostic delay concerned colon cancer, in 27 cases rectal cancer and in one case the diagnosis was squamous cell cancer. At the time for diagnosis 11 patients with colon cancer had metastases and in cases with rectal cancer this figure was 12. The two cases with a delay in recognizing recurrent/metastatic disease were cases of rectal cancer. Of these 23 patients with metastatic disease the grading of the primary CRC cancer according to the classical Dukes’ method was Dukes’ B in 1, Dukes’ C in 10 and in 12 cases no grading could be identified as no histomorphological analyses had been performed.

The sex and age distribution in six cohorts of the claimers on the date of missed diagnosis is depicted in Table 1. Mean and median age of the patients in the study was 59 years.

**Table 1:** Age distribution and gender for individuals at the time of erroneous diagnosis of colorectal cancer

**Age (years)**	**Females (number)**	**Males (number)**	**Total**
≤ 40	1	2	3
41-50	7	4	11
51-60	4	14	18
61-70	13	7	20
71-80	4	4	8
≥ 80	1	1	2
Total	30	32	62

Haematoschisis or anaemia was the predominating symptom in 33 cases (53 %), changed bowel habits in six, and pain in five cases. In 14 patients the different symptoms overlapped (Table 2). In two patients there was an error in the follow up after curative surgery. Potential causes of claims at the time of missed diagnosis are shown in Table 3. In most cases clinical symptoms failed to be recognized by the first doctor seen by the patient (44/62). In ten cases the diagnostic delay was due to incomplete clinical examination (colonoscopy, barium enema, CT). In four cases the delay was due to radiological error.

**Table 2:** Principal presenting symptom in patients for whom the diagnosis of colorectal cancer was delayed

**Symptoms**	**No. of patients**	**No. of patients ≤ 50 years**	**No. of patients > 50 years**
Bleeding and/or anaemia	33	6	27
Diarrhoea	6	2	4
Pain	5	1	4
Renal symptoms	1	0	1
Perineal infection	1	0	1
Overlapping symptoms	14	5	9
Recurrent disease	2	0	2
Total	62	14	48

**Table 3:** Potential causes of diagnostic delay in 62 patients with colorectal cancer

**Symptoms**	**No. of patients**
Clinical error Clinical signs were not recognized	44
Delay in appropriate investigation	4
Incomplete clinical examination	10
Radiological error	
Misinterpretation of malignant lesion	2
Incomplete X-ray	2
Total	62

Localisation of the cancer in the colon or the rectum and staging of CRC according to Dukes’ classical criteria and the duration of diagnostic delay of more or less than 6 months are shown in Table 4.

**Table 4:** Delay time in relation to type of primary tumour and staging at diagnosis

**Delay time (months)**	**Dukes’ A-B**	**Dukes’ C**	**Dukes’ D**	Total*
< 6	8	15	8	31
≥ 6	5	9	15	29
Total	13	24	23	60
**Delay time (months)**	**Dukes’ A-B**	**Dukes’ C**	**Dukes’ D**	Total*
< 6	8	15	8	31
≥ 6	5	9	15	29
Total	13	24	23	60

There were 23 patients who had metastases (Dukes’ D) at the time of diagnosis. Eleven of these patients had liver metastases, three lung metastases, one both liver and lung metastases, two skeleton metastases, one ovarian, one suprarenal and four generalized cancer.

The median alleged delay in diagnosis of CRC was 6.0 months (range 0.7-43). In five claims no delay was identified and in one the delay was less than one month. The majority of patients who had a delay of less than 6 months (24/32) were without metastatic disease. For 17 claimants the diagnostic delay was more than one year, eight of them had metastatic disease. The claimed diagnostic delay was considered to have had an impact on the extent of therapy concerning the surgical, radiation or chemotherapeutic procedures in 12 of 60 patients (20 %). Five of these patients had metastases and eight of them had a delay of more than 6 months (Table 5).

**Table 5:** The impact of the diagnostic delay on therapy

**Stage**	**yes (number)**	**probably (number)**	**no (number)**	Total (number)*
Dukes´A or B	1	0	12	13
Dukes´C	6	0	18	24
Metastatic disease “Dukes´D”	3	2	18	23
Total	10	2	48	60

* In one case no cancer was found after preoperative irradiation and one patient had a squamos cell cancer.

In seven patients and probably in an additional two, in total 9 of 62 (15 %) the diagnostic delay was considered to have had an impact on prognosis. Three of the patients had metastatic disease at diagnosis. In eight of these cases the diagnostic delay was more than 6 months and in one less than 6 months. Of the 29 patients with a delay of more than 6 months, 8 were considered to have experienced a worse prognosis. Of the 31 cases with a delay of less than 6 months, this figure was one (p = 0.0158).

The Kaplan-Meier curves showed no significant difference in survival related to duration of diagnostic delay of more or less than 6 months (Figure 1) or 12 months. There was a significant difference in survival (p < 0,0001) between patients with and without metastatic disease at diagnosis (Figure 2).

Economic compensation for diagnostic delay was approved for 49 of 62 patients (79 %). The rules stated for economic compensation are that there should be a causal relationship between the claimed injury and the care and that it could be established that the injury could have been avoided.

## Discussions

This retrospective study identified a low number of claims for compensation for patients who reported a diagnostic delay of CRC related to the clinicians (53/62) or to the providers (9/62). This number covered a period of 14 years, i.e. approximately five claims annually compared with an overall rate of 6200 new cases of CRC reported each year to the Swedish Cancer Register. More than half of the claims (52 %) were reported by patients younger than 60 years, who account for only 20 % of the age distribution of patients with CRC. The majority (53 %) of diagnostic errors were related to a history of gastrointestinal bleeding or anaemia and in these cases no diagnostic procedures were carried out or incorrect procedures were undertaken. At diagnosis more than one third (37 %) had an advanced cancer with metastatic disease.

From the present study it is not possible to draw any conclusion of the total rate of diagnostic delay of CRC in Sweden. It can only be stated that during the period studied the malpractice insurance company (LÖF) processed about five cases annually dealing with diagnostic delay of CRC. This may be an underestimate because some patients may refrain from filing claims. However, these data correspond rather well with those reported by the Physician Insurers Association of America (PIAA). PIAA, which was started in 1985, has received about 2500 colorectal claims between 1985 and 2006; e.g. about 120 claims per year or one per 1000 new cases of CRC in clinical care in the US (150.000 new cases are diagnosed yearly). There was, however, a big difference between the two insurance systems regarding economic compensation. In the Swedish system almost 80 % were compensated, in the US system somewhat more than 25 % [5].

According to *Eurocare 4* the 5-year survival is 61.5 % for patients diagnosed between 2000 and 2002 [6]. In Sweden the relative survival rate is slightly higher for women than for men, but almost equal for patients if the tumour is located in the colon compared with that located in the rectum.

It is a dogma that early diagnosis of cancer before the onset of symptoms improves survival [7,8]. However, the importance of delay when symptoms have appeared is unclear. That was obvious from a meta-analysis including 40 studies of CONCORD representing 20,440 patients. Of the 26 studies that were evaluated, 20 showed no association between delay and survival, four showed that delay contributed to a better prognosis, and two studies to a poorer prognosis [9]. The results of this review suggest that there is no clear association between diagnostic or therapeutic delay and survival in CRC patients. In contrast with these statements, it was shown in a Danish study of 740 patients that a total therapeutic delay of at least 60 days had an impact on long-term survival on patients with rectal cancer but that there was no association between delay and mortality for patients with colonic cancer [10]. It was also apparent from this study that neither provider delay, nor hospital delay had any influence on survival; the only factor that had a negative effect was the total therapeutic delay and that was restricted to patients with rectal cancer. The observation that diagnostic delay only relates to rectal cancer and not to colonic cancer has been described in other studies [11,12].

This study indicated that the delay of some kind affected the therapy since the delay resulted in a need for more extensive surgery or for down-sizing X-ray or chemotherapy. For patients with metastatic disease 5 out of 23 were not subjected to liver surgery and/or down-sizing chemotherapy due to too advanced disease. It was suggested that the diagnostic delay had an influence on the prognosis in only 15 % of the patients; all these were referred to stage Dukes’ C (n = 6) or metastatic disease (Dukes’ D) (n = 3). These figures are based on personal experience of the authors and are open for debate. It can, however, be considered obvious that diagnostic delay has a negative influence on a cancer disease but the analysis made here on the basis of our study provides support for those who question this statement [13,14]. It is undebatable that any cancer has the potential to metastasize before it is clinically detectable [15].

In the present study there was no significant impact on survival of a delay, whether the delay was more than 6 months or less than 6 months. The most important impact on survival in this selected group of patients with CRC was that metastatic disease of any location significantly (p < 0,001) reduced survival (Figure 2). These findings have to be seen against in the context of those patients who claimed diagnostic delay had metastatic disease in 37 %.

The relation between diagnostic delay and survival is complex. Findings that raise questions about standard cancer kinetics come from studies that have found that longer delay has resulted in improved survival [14]. The most common explanation for this observation is that less aggressive cancers have a longer lead time until they produce significant symptoms [10]. Cancer-mortality statistics are used for comparisons but they are influenced by well-known problems of confounding factors that can explain the varying results [16].

In this study the diagnostic delay was in 71 % of the cases related to the inability of the doctor who first met the patient to identify adequate signs of CRC, i.e. bleeding, anaemia and changes in bowel habits. The diagnostic guidelines established in Sweden were clearly not followed. Colonography or colonoscopy (and biopsy, if indicated), must be considered as routine examinations with a diagnostic predictive value of above 90 and 80 %, respectively [17]. In a few cases the delay was caused by misinterpretation of X–ray investigations. It is important to emphasize that when a barium enema is used and is difficult to interpret, a colonoscopy should always be performed.

The insurance company (LÖF) showed a generous attitude to the claimers, since it is documented that in 79 % of the case the patients were economically compensated. The high rate can be explained by the fact that LÖF covers injuries where it can be proved that the injury is related to the medical care and could have been avoided. There are no regulations requiring disciplinary actions in LÖF´s system but the insurance company is working on developing objective grounds for determining the right for the patient to receive compensation according to the provisions of The Swedish Patient Injury Act.

The strength of this analysis is related to the condition that the data were collected from a well-established patient national insurance system in Sweden covering a long period (14 years). The information was based both on the hospitals’ records and on patients´ reports to LÖF. The survival data were obtained from an authoritative organization on a national basis. The limitations of this study arise because of the nature of retrospective analyses associated with sometimes incomplete data and a limited number of patients. Furthermore, the evaluation of impact concerning prognosis and consequences of the treatment and course was based on the joint judgments of the authors independent of the primary statements made by the insurance company.

## Conclusions

This study indicated that patients with CRC rarely file claims for economic compensation for diagnostic delay. The delay of diagnosis was a median of six months and was mainly the result of clinical errors. The delay was considered to have had an impact on treatment in a fifth of the patients who filed claims and the prognosis was postulated to have been adversely affected in 15 %. No impact on survival was identified. Economic compensation was given to the claimers in almost 80 %.

## Competing interests

Two of the authors (LOH, HJ) are medical experts in the Swedish Patient Claims Panel and one (JA) is permanently employed by The County Council´s Mutual Insurance Company as medical director. There are no competing financial interests.

## Authors’ contribution

All three authors designed the study design. Two authors (LOH and HJ) scrutinized the claims and the charts. The analysis of data, drafting of first manuscript was done by the three authors. The final version of this manuscript was read and approved by the three authors.
